# An overview of host immune responses against *Leishmania spp.* infections

**DOI:** 10.1093/hmg/ddaf043

**Published:** 2025-04-27

**Authors:** Hanna Paton, Prabuddha Sarkar, Prajwal Gurung

**Affiliations:** Inflammation Program, University of Iowa, 431 Newton Road, Iowa City, IA 52242, United States; Department of Internal Medicine, University of Iowa, 431 Newton Road, Iowa City, IA 52442, United States; Immunology Graduate Program, University of Iowa, 431 Newton Road, Iowa City, IA 52242, United States; Inflammation Program, University of Iowa, 431 Newton Road, Iowa City, IA 52242, United States; Department of Internal Medicine, University of Iowa, 431 Newton Road, Iowa City, IA 52442, United States; Inflammation Program, University of Iowa, 431 Newton Road, Iowa City, IA 52242, United States; Department of Internal Medicine, University of Iowa, 431 Newton Road, Iowa City, IA 52442, United States; Immunology Graduate Program, University of Iowa, 431 Newton Road, Iowa City, IA 52242, United States; Interdisciplinary Graduate Program in Human Toxicology, University of Iowa, 431 Newton Road, Iowa City, IA 52242, United States; Center for Immunology and Immune Based Disease, University of Iowa, 431 Newton Road, Iowa City, IA 52242, United States; Iowa City Veterans Affairs (VA) Medical Center, 601 US-6, Iowa City, IA 52246, United States

**Keywords:** Leishmania, Parasite, Immune response, Host-pathogen interaction

## Abstract

*Leishmania spp.* infections pose a significant global health challenge, affecting approximately 1 billion people across more than 88 endemic countries. This unicellular, obligate intracellular parasite causes a spectrum of diseases, ranging from localized cutaneous lesions to systemic visceral infections. Despite advancements in modern medicine and increased understanding of the parasite’s etiology and associated diseases, treatment options remain limited to pentavalent antimonials, liposomal amphotericin B, and miltefosine. A deeper understanding of the interactions between immune and non-immune cells involved in the clearance of *Leishmania spp.* infections could uncover novel therapeutic strategies for this debilitating disease. This review highlights recent progress in elucidating how various cell types contribute to the regulation and resolution of *Leishmania spp.* infections.

## Introduction

*Leishmania spp.* infection causes a debilitating disease known as leishmaniasis. *Leishmania spp.* are obligate intracellular parasites that are transmitted by the bite of sandflies—primarily *Phlebotomus* and *Lutzomyia spp.*, although several other species have been reported as well [1]—and endemic in several countries throughout the world [2]. Currently, the disease can be found in 99 countries, with close to 300 000 new cases reported worldwide [2]. Given that most of the people affected with leishmaniasis are found in third world countries without access to proper healthcare, the reported cases are highly underestimated, and the real incidence is probably much higher. Disease caused by *Leishmania spp.* can manifest as cutaneous, mucocutaneous and visceral forms. Cutaneous and mucocutaneous leishmaniasis (CL and ML) are often self-healing, although these can leave life-altering scars in affected tissues and have social stigma associated with it. Visceral leishmaniasis (VL), also known as kala-azar, affects multiple organs leading to enlarged spleen and liver. Unlike cutaneous and mucocutaneous forms, visceral disease if untreated are fatal to the host. Despite these drastic outcomes of the parasitic infections, and the sheer number of people that are affected worldwide—leishmaniasis is still considered a neglected tropical disease by world health organization [2]. Thus, the treatment strategies to fight leishmaniasis are still limited to our use of few drugs that include Amphotericin B, Miltefosine and Antimonial [2, 3]. Unfortunately, the compliance to these drugs have been limited because of their adverse side effects, and long treatment course.

More than 20 different *Leishmania spp.* are known to cause disease in humans [1]. *Leishmania spp.* are broadly divided into old-world and new-world species based on where these parasites originated and are prevalent. The old-world species including *Leishmania major*, *Labrus donovani*, *L. infantum* and *L. tropica*—all originated from eastern hemisphere, specifically Africa, Asia and Middle East [1, 4]. On the other hand, *Leishmania spp.* such as *L. amazonensis*, *L. mexicana* and *L. braziliensis* found in the western hemisphere—specifically Mexico, Central America and South America—are considered new-world disease [1, 4].

The disease manifestations following *Leishmania spp.* infections are dictated by the species of *Leishmania* infecting the host [5]. *Leishmania spp.* such as *L. major*, *L. braziliensis*, *L. amazonensis* are the common cause of CL. *L. braziliensis*, *L. infantum* and *L. mexicana* are known to cause ML. *L. donovani* and *L. infantum* are known to cause VL. The immune mechanisms underlying the tropism and manifestations of different disease with specific *Leishmania spp.* are still unclear. Some of the general thoughts for why some species causes cutaneous but others cause visceral diseases have been linked with—1) affinity of *Leishmania* parasites to infect different tissue specific phagocytic populations and establish infection, 2) temperature sensitivity of different *Leishmania* parasites whereby parasites that cause visceral infections thrive in temperatures close to body temperature, 3) external factors that include differential ability of sandfly saliva to promote vasodilation, which can subsequently affect visceralization, and 4) genetic backgrounds of the host [6].

Despite the advancement in our understanding of host responses to *Leishmania spp.* infections, vaccines and specific therapeutics for the treatment of this debilitating disease are still missing. Here, we review the literature from the perspective of different cells that interact and respond during *Leishmania spp.* infection to either provide protection or propagate disease.

## Keratinocytes

**Figure 1:**
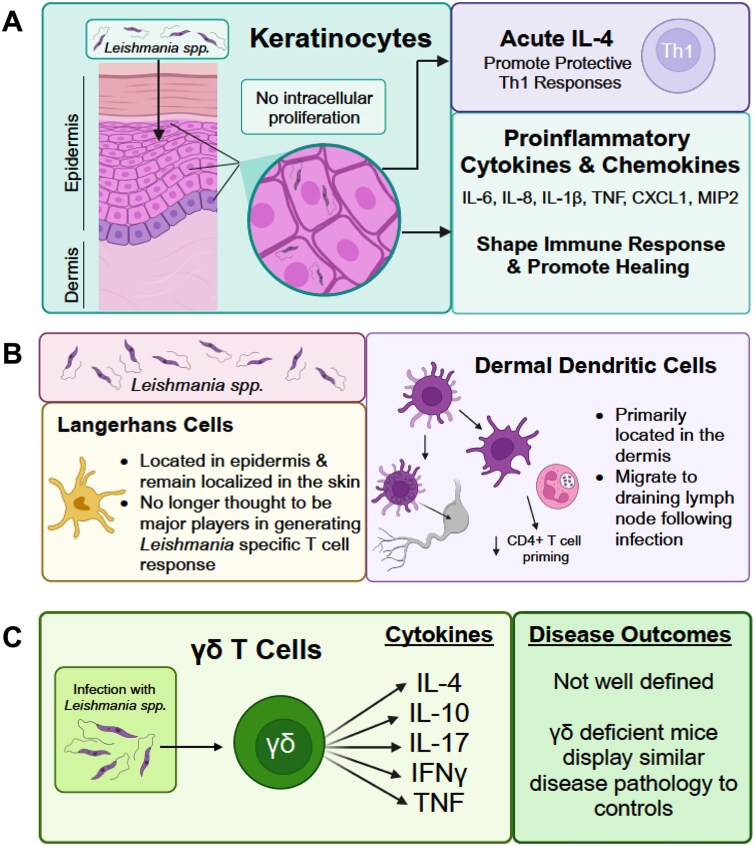
*Leishmania* sensing in the skin. Skin resident cells that include keratinocytes, Langerhans cells, and γδ T cells are the first line of defense following infection with *Leishmania* spp. (A) Keratinocytes can harbor parasite and secrete cytokines—To both shape the immune response and promote healing. Of key importance is an acute burst of keratinocyte-derived acute IL-4, shown to promote Th1 responses. (B) Langerhans cells and dermal dendritic cells, play distinct roles in antileishmanial immunity. (C) γδ T cells have been shown to secret several cytokines that include IL-4, IL-10, IL-17, IFN-γ and TNF—However, cytokines from these gd T cells in disease control remain unclear.

Keratinocytes are one of the primary immune cell types in the skin epidermis and are among the first cells to encounter *Leishmania spp.* during infection (Fig. 1A)**.** The role of keratinocytes in modulating immune responses is only beginning to be understood. Given that *Leishmania spp.* are obligate intracellular parasites, keratinocytes have been proposed as potential host cells for these parasites. Studies by Ronet et al. and Scorza et al. demonstrated that approximately 1%–3% of keratinocytes become infected with *Leishmania spp.* parasites [7, 8]. Considering that keratinocytes constitute 90%–95% of the cells in the skin, even an infectivity rate of 1%–3% could significantly affect disease outcomes. Interestingly, keratinocytes do not support the intracellular replication of internalized *Leishmania* parasites [8].

Importantly, keratinocytes produce various cytokines and chemokines, such as CXCL1, MIP2, LIX, IL-6, and IL-8, during *Leishmania spp.* infection. These factors can instruct immune cells and promote healing responses [7, 8]. Moreover, supernatants from *Leishmania*-infected PBMCs have been shown to induce keratinocyte death, which can influence inflammation, local immune responses, and lesion development [9]. Mechanistically, this keratinocyte death is dependent on Fas–FasL and TRAIL-TRAILR interactions, as keratinocytes express FasL and TRAILR [9].

A study by Ehrchen et al. revealed that keratinocytes are a major source of early cytokines during *Leishmania spp.* infection [10]. Analysis of gene and protein expression in 16-h *L. major*-infected skin indicated increased levels of several pro-inflammatory cytokines, including IL-1β, IL-4, IL-6, and TNF, in resistant C57BL/6 mice. Similar gene and protein expression changes were observed in human keratinocytes exposed to *L. major* and *L. infantum*. Notably, neutralization of early IL-4 or genetic deletion of IL-6 in the non-immune compartment (possibly in keratinocytes) during *L. major* infection in C57BL/6 mice resulted in reduced IFN-γ production, increased parasite burden, and enhanced footpad swelling [10]. These findings highlight the critical role of early pro-inflammatory cytokines—specifically IL-4 and IL-6—produced by keratinocytes in shaping subsequent protective immune responses during *L. major* infection. Subsequent studies demonstrated that IL-4 signaling back onto keratinocytes is dispensable for generating protective immune responses. This was shown through experiments using keratinocyte-specific deletion of IL-4R, i.e. *Il4ra*^flox/flox^ x *Krt14*^Cre^ mice [11, 12].

Altogether, these studies highlight the critical role of keratinocytes as potential reservoirs for *Leishmania spp.*, although their roles in supporting or eliminating these intracellular parasites require further investigation. External factors, such as death ligands including Fas and TRAIL, can induce keratinocyte death during *Leishmania spp.* infection. This process not only limits intracellular parasite growth within keratinocytes but also propagates inflammation. These interactions further prompt keratinocytes to produce pro-inflammatory cytokines in the local microenvironment, shaping subsequent protective immune responses at later stages. It is evident that keratinocytes play a pivotal role during *Leishmania spp.* infection. Future studies should focus on examining specific pathways within keratinocytes and their crosstalk with other immune cells.

## Langerhans and dermal dendritic cells

Within the skin, a distinct population of dendritic cells known as Langerhans cells (LCs) resides in the epidermis and has long been implicated in the initial dissemination of *Leishmania* parasites (Fig. 1B). Although LCs are currently classified as a subset of dendritic cells, they possess unique properties that differentiate them from other dendritic cell populations, including dermal dendritic cells. LCs are distinguished from dermal dendritic cells by the presence of Birbeck granules, a characteristic ultrastructural feature [13]. In contrast, dermal dendritic cells constitute a heterogenous population within the dermis, which remains incompletely characterized [14, 15]. Studies suggest that distinct DC populations at the site of infection can profoundly influence the differentiation and phenotype of CD4 T helper cells [16].

The migration of dendritic cells from CL lesions to the draining lymph node is believed to be essential for generating an effective CD4 T cell response and achieving parasite clearance [17]. Using adoptive transfer models, it has been demonstrated that *L. amazonensis*-infected dendritic cells exhibit impaired migration to the draining lymph node [18]. Initially, LCs were proposed to phagocytose *Leishmania* parasites *in vivo* and migrate to draining lymph nodes to facilitate antigen presentation and T cell activation [19, 20]. However, subsequent studies have ruled out LCs, and revealed that the primary migratory dendritic cell population in CL consists of langerin-negative, CD8α-negative dendritic cells, which exhibit heterogenous expression of DC- and skin-associated markers [21, 22].

Beyond antigen presentation, dermal dendritic cells have been shown to mediate neutrophil recruitment in cutaneous bacterial infections [23]. While direct evidence of dermal dendritic cell function in leishmaniasis remains limited, available data suggest they play a critical role in early-stage anti-*Leishmania* immunity. During *L. major* infection, dermal dendritic cells were observed capturing infected neutrophils. Moreover, dermal dendritic cells from neutrophil-depleted mice exhibited increased activation marker expression and enhanced antigen presentation capacity ex vivo, leading to improved CD4 T cell priming *in vivo* [24].

## γδ T cells

γδ T cells were first isolated by subtractive hybridization from a cDNA library in 1984 by Tonegawa and colleagues using an IL-2-dependent cytotoxic T cell clone [25–28]. Initially designated as γ-TCR genes consisting of V-, J-, and C-region elements, γ-TCR are expressed on the plasma membrane and associate with CD3 receptors [26]. Subsequently, Brenner et al discovered another T3 glycoprotein subunit termed δ-TCR, which was proposed to form a heterodimer with γ-TCR (i.e. γδ TCR) analogous to the αβ TCR complex [29]. When activated in-vitro, cells expressing γδ-TCR exhibit MHC independent cytolytic activity, as well as recognize wide arrays of foreign and endogenous antigens [27, 30, 31].

One of the first efforts were made to detect γδ T cells in leishmania-infected BALB/c and C57BL/6 mice back in 1991 [32]. Unfortunately, these studies failed to detect γδ T cells in the lesion-draining lymph nodes despite earlier proposals that γδ T cells are one of the first line of defenses during infections [32, 33]. Importantly, γδ T cell deficient mice showed no defects in clearance of *L. major* infection when compared to C57BL/6 mice [34]. In contrast, αβ T cell deficient mice developed non-healing infections [34].

To the contrary, kinetic analysis of γδ T cells in *L. major* infected susceptible BALB/c and resistant CBA/J mice revealed expansion of these cells in the spleen and lymph node [35]. In another study, expansion of γδ T cells during *L. major* infection was reported to be dependent upon lymphokines specific for Th2 type CD4 T cells [36]. Later a study suggested γδ T cells as a potential source of IL-4 in *L. major* infected BALB/c mice [37]. γδ T cells can produce IL-17 as well, which is important for immune suppression during the early stage of *L. donovani* infection in C57BL/6 mice [38]. Likewise, Terrazas et al showed that γδ T cells produce IL-17A responsible for susceptibility of C57BL/6 mice during *L. donovani* infection [39]. More recently, γδ T cells were demonstrated to produce IL17A during *L. amazonensis* infection in susceptible Sv129 mice [40]. Production of IL-17 by these γδ T cells was associated with pathogenesis during cutaneous leishmaniasis [40].

Apart from rodents, elevated γδ T cells were also reported in the case of visceral leishmaniasis in dogs of various breeds, compared to the healthy controls—although, the exact role of these cells remain unclear [41]. In humans, γδ T cells were reported to be increased in patients suffering from both cutaneous and visceral leishmaniasis [42–44]. Even though the exact role of γδ T cells during leishmania infection remain to be characterized in humans [45], γδ T cell from visceral leishmaniasis patient were shown to express IL-10, TNF and IFN-γ. Thus, these cells were speculated to have a cytotoxic as well as immuno-regulatory role during visceral leishmaniasis [46]. Similarly, production of IL-10 by CD4-CD8 double negative γδ T cells in PBMCs following incubation with soluble leishmania antigen in cutaneous leishmaniasis patients was also reported in another study. This also indicates an immuno-regulatory role of these cells during leishmaniasis [45].

In this section we have reviewed the role of γδ T cells during different leishmania infection in human and mice (Fig. 1C). Though the exact role of these cell types during leishmania infection remains to be confusing, but reports suggest that the significance of the γδ T cells depends more on the type of cytokines they secrete during leishmania infection.

## Mast cells

**Figure 2:**
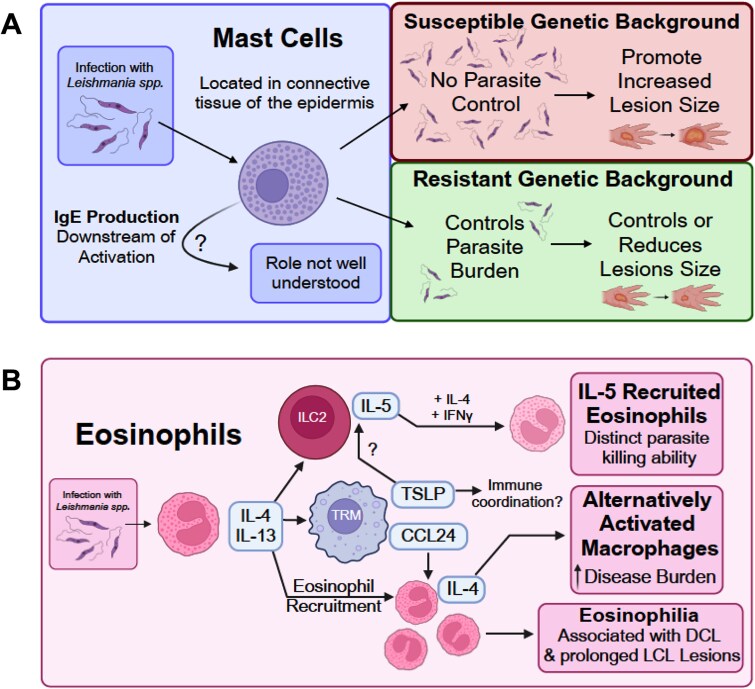
Roles of mast cells and eosinophils in disease control during *Leishmania* infection. (A) Mast cells present in the epidermis have been shown to mediate both susceptibility and resistance, contingent on genetic background. (B) Eosinophils can promote susceptibility to disease via continued secretion of IL-4 to promote alternative activation of macrophages. Eosinophilia, or the accumulation of eosinophils, at the site of infection, has been associated with more severe and prolonged disease. More recently, a protective role of eosinophils has come to light via their crosstalk with tissue resident macrophages and type 2 innate lymphoid cells (ILC2) in the skin.

Mast cells are innate immune cells abundantly present in connective tissues, including the skin. While mast cells are primarily known for their role in IgE-mediated hypersensitivity reactions and anaphylactic shock, their involvement in innate immune responses is equally significant, as evidenced by their critical role in producing inflammatory cytokines [47]. The role of mast cells during *Leishmania spp.* infections has been contentious, and whether these cells play a protective or pathogenic role remains unresolved (Fig. 2A).

An earlier study investigating the role of mast cells used *W/W*^V^ and *Sl/Sl*^d^ mice, both deficient in mast cells, and found that the absence of mast cells rendered mice resistant to *L. major* infections [48]. Mast cell-deficient *W/W*^V^ and *Sl/Sl*^d^ mice developed significantly smaller lesions compared to their littermate controls. However, mast cell deficiency did not affect parasite titers [48]. Reconstitution of *W/W*^V^ mice with mast cells reversed this resistance and led to larger lesions [48]. Similarly, a more recent study examining the role of mast cells in susceptible BALB/c mice showed that *W/W*^sh^ mice (deficient in mast cells) developed significantly smaller lesions compared to their littermate controls following *L. major* infection [49]. In this case, the protection observed in *W/W*^sh^ mice correlated with better parasite clearance, and reconstitution of mast cells reversed the protection.

In contrast, Dudeck et al reported that mast cell-deficient *W/W*^sh^ mice on a resistant C57BL/6 background developed larger lesions than C57BL/6 mice [50]. This increased susceptibility was associated with higher parasite burden, increased Th2 responses, and reduced Th1 responses [50]. One limitation of this study was the use of non-littermate C57BL/6 mice as controls, which complicates interpretation of the actual role of mast cells. Nonetheless, similar protective roles for mast cells were observed in the same group’s study using *W/W*^V^ mice [51]. In this case, reconstitution of *W/W*^V^ mice with mast cells reversed the disease course, resulting in smaller lesions and reduced parasite burden.

Studies using mast cell-deficient models highlight both protective and pathogenic roles for mast cells during *Leishmania spp.* infections. The discrepancies observed may be attributed to differences in genetic background (resistant versus susceptible mouse strains); however, further comparative studies are needed to fully elucidate the dual roles of mast cells in leishmaniasis.

## Eosinophils

Eosinophils—granulocytes that are primarily associated with parasitic infections and allergies [52]—play a critical role in the immune response to *Leishmania* spp. infections (Fig. 2B). Effective control and clearance of *Leishmania* spp. rely on a robust type 1 pro-inflammatory response [53, 54]. Conversely, type 2 inflammation, characterized by its associated cytokines, is known to promote parasite persistence and exacerbate disease severity [55].

In CL, eosinophils have been identified as the primary producers of the type 2 cytokine IL-4 [56, 57]. IL-4 facilitates the recruitment of eosinophils to infection sites and promotes the alternative activation of macrophages (M2 macrophages) [53, 56], which are associated with more severe disease progression [55]. Previous studies demonstrated that ex vivo stimulation of tissue-resident macrophages (TRMs) increases the production of the eosinophil-attractant chemokine CCL24, amplifying eosinophil recruitment [56]. Recent findings also highlight the impact of eosinophil-derived IL-4 and IL-13 on outcomes of *L. major* infections. A novel alarmin circuit involving TRMs, type 2 innate lymphoid cells (ILC2s), and eosinophils has also been identified. In this circuit, ILC2s act as the primary producers of IL-5 in the skin [58].

Interestingly, eosinophils recruited by IL-5 display distinct parasite-killing abilities when exposed to IL-4 and IFN-γ [59]. Transgenic IL-5 mice (C3H/HeN-TgN(IL-5)-Imeg)—in which 50% of peripheral blood immune cells are eosinophils—show high resistance to infection with *L. amazonensis* [60]. Similarly, partial resistance to *L. amazonensis* was observed in highly susceptible BALB/c mice electroporated with plasmids encoding IL-4 or IL-5, with IL-5 providing a greater degree of protection [60]. These observations underscore the critical roles of IL-5 and eosinophils in parasite killing and the immune response in CL.

ILC2s production of IL-5 is highly activated by alarmins [61], such as epithelial cytokines IL-25, IL-33, and thymic stromal lymphopoietin (TSLP), which coordinate skin inflammation [62]. While alveolar macrophages were previously the only TRMs known to produce alarmins like IL-33 during infection [62], recent research shows that dermal TRMs produce TSLP—but not IL-25 or IL-33—following *L. major* infection [57]. Moreover, selective deletion of eosinophil-derived IL-4 and IL-13 reduces dermal TRM and eosinophil-attractant gene expression, as well as IL-5 production by ILC2s [57]. These findings suggest that the ILC2-TRM-eosinophil signaling axis is a potential predictor of susceptibility to CL, influencing whether the infection remains localized or becomes diffuse.

In clinical settings, eosinophils have been identified at inflammation sites in CL patients [63]. A study of patients with *L. mexicana* infections revealed increased eosinophilia, particularly in cases of diffuse CL (DCL) and prolonged localized CL (LCL) [63]. Additionally, eosinophil phenotypes and cytokine profiles differed between DCL and LCL patients, further implicating eosinophils in the progression, dissemination, and chronicity of the infection.

## Neutrophils

**Figure 3:**
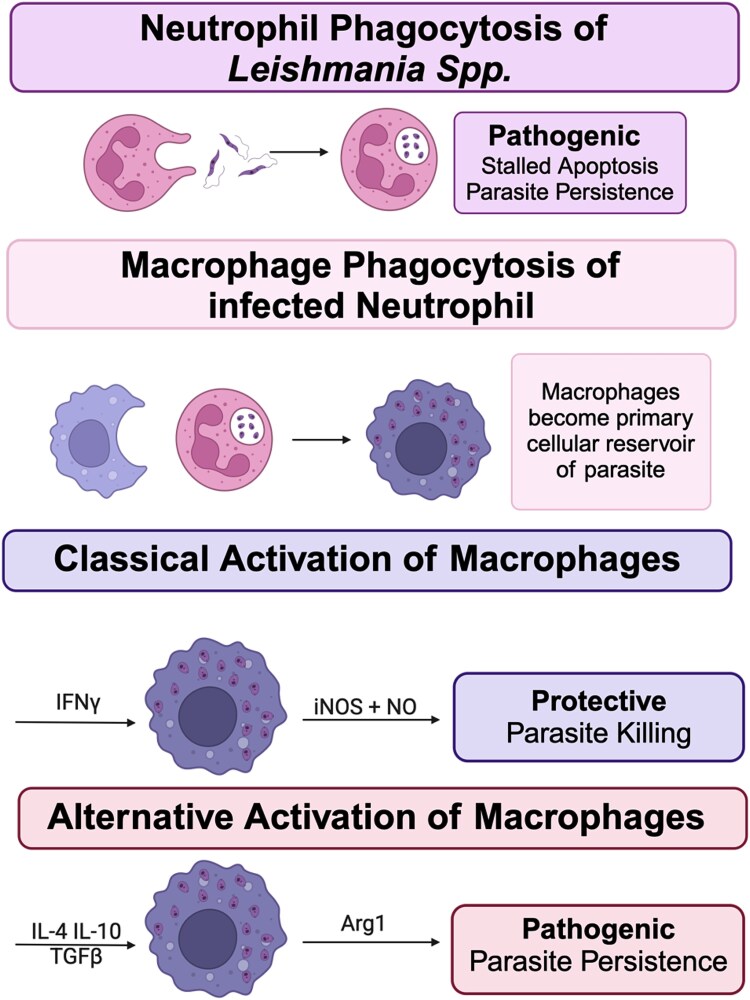
Neutrophils and macrophages in leishmaniases. Both neutrophils and macrophages play key roles in establishment and resolution of leishmaniases. Neutrophils: Neutrophils phagocytose *Leishmania spp*. and temporarily become the primary cellular reservoir for *Leishmania spp.* soon after, macrophages phagocytose infected neutrophils and debris to become the primary cellular reservoir of *Leishmania spp.* macrophages: After becoming the primary cellular reservoir of *Leishmania spp.*, route of macrophage activation determine parasite killing vs. persistence. Classical activation (M1 polarization) of macrophages results parasite killing and protection, while alternative activation (M2 polarization) is pathogenic and results in parasite persistence.

Neutrophils are key players in innate immunity, acting as first responders to infection and injury by rapidly phagocytosing pathogens to prevent their dissemination [64, 65]. Following a sandfly bite, dermal injury induces localized inflammation and blood pooling, triggering the swift migration of neutrophils to the site of infection [65, 66]. Within the infected tissue, neutrophils activate their defense mechanisms. However, *Leishmania* parasites have evolved strategies to subvert and exploit neutrophil responses, influencing antileishmanial immunity [67] (Fig. 3).

Neutrophils phagocytose *Leishmania* during initial infection stages, supporting the ‘Trojan horse hypothesis,’ where parasitized neutrophils act as vectors for macrophage infection [68, 69]. *In vitro* studies with *L. major* show that infected neutrophils exhibit prolonged lifespans (up to two days) and secrete CCL4, a chemokine critical for recruiting monocytes and macrophages [67, 70]. While neutrophils produce reactive oxygen species (ROS), these are insufficient to kill *Leishmania* parasites [71]. Instead, the parasites benefit from extended neutrophil survival to optimize transfer to macrophages.

Concurrent with neutrophil recruitment, the *Leishmania* parasites exploit the C3 complement cascade [72–74]. Parasite virulence factors such as gp63 and SPF-1 inhibit efficient cleavage of C3, leading to incomplete membrane attack complex (MAC) formation [75–77]. This results in parasites opsonized with inactivated C3b (iC3b), facilitating their phagocytosis by neutrophils and skin-resident phagocytes [72, 78, 79]. Once internalized, parasites persist in phagolysosomes by blocking NADPH oxidase (NOX2) assembly, essential for generating antimicrobial ROS [66, 80].

Surface lipophosphoglycan (LPG) expression by *L. donovani* blocks NOX2 assembly in macrophages [80]. In neutrophils, however, NOX2-generated ROS appear more critical for inflammation control than for parasite elimination [81]. Disruption of NOX2 activity alters neutrophil cell death from apoptosis to necrosis, potentially increasing inflammation and tissue damage while promoting parasite dissemination [81]. Recent advances in the field of cell death have revealed the complexity and interplay of various cell death pathways, including apoptosis, pyroptosis, necroptosis and NETosis during *Leishmania* infection [82]. Study by Kanneganti and colleagues demonstrated that most infectious stimuli activate a distinct cell death pathway termed PANoptosis [83]. PANoptosis is a distinct innate immune cell death pathway initiated by innate immune sensors and driven by caspases and RIPKs through multiprotein PANoptosome complexes. While PANoptosis shares molecular components with apoptosis, pyroptosis, and necroptosis, it is mechanistically distinct from these cell death pathways— and the deletion of core components of these pathways does not block PANoptosis [84, 85], and the formation of a PANoptosome complex is a unique feature which has been visualized in individual cells by microscopy [85–88]. However, our understanding of how PANoptosis is triggered during Leishmania infection remains limited, and further studies directly addressing this topic are needed.

Further investigation with *L. major* has implicated NOX2-dependent ROS production in inducing a ‘paused apoptotic’ neutrophil phenotype following C3/CR3-mediated internalization. While the uptake of *Leishmania* parasites via C3/CR3 has been well-characterized in other phagocytes, its role in neutrophils was not previously established [66]. Recent evidence now links the uptake of iC3b-opsonized *L. major* through complement receptor 3 (CR3), also known as CD11b or MAC-1, directly to the NOX2-dependent respiratory burst in neutrophils [66, 81]. *C3*^−/−^ mice exhibit an overall reduction in lesion size but show no significant impairment in their ability to establish chronic infections. In contrast, CR3 (*Cd11b*^−/−^) deficient mice demonstrate a larger decrease in the frequency of infected neutrophils compared to *C3*^−/−^ mice, despite showing similar neutrophil numbers 90 minutes post-infection with *L. major* [66, 81]. While *C3*^−/−^ neutrophils show no significant difference in parasite internalization, *in vitro* experiments reveal a threefold reduction in *L. major* uptake by CR3-deficient neutrophils. This indicates that although C3 is not essential for parasite internalization, it enhances the efficiency of the process. Furthermore, some disease pathology may stem from dysregulated inflammation rather than parasite load.

*Leishmania* appears capable of altering neutrophil cell death fate to avoid immune detection, particularly during early infection stages. Infected neutrophils show downregulation of protein and gene expression for apoptosis-associated caspases, such as caspase-3, both *in vivo* and *in vitro* [66]. This modulation of cell death mechanisms may allow *Leishmania* to evade immune responses and establish infection.

*Leishmania spp.* also evades neutrophil extracellular traps (NETs), a specialized form of neutrophil cell death involving DNA extrusion. While NETs enhance localized inflammation, they largely fail to kill *Leishmania spp.* [89, 90]. This increased, non-pathogen-specific inflammation is associated with tissue damage and further dissemination of the disease [91]. The *Leishmania spp.* virulence factor, inhibitor of serine peptidase-2 (ISP2), plays a significant role in modulating susceptibility to NETosis by inhibiting the proteolytic activity of NETs. Infection with D *Isp2/3 L. major* results in reduced neutrophil phagocytosis but enhanced NE-mediated parasite uptake by macrophages [89, 92]. Interestingly, recent findings suggest a correlation between genetic susceptibility and the capacity of ex vivo NETs to kill *Leishmania* promastigotes *in vitro*. However, the expression of ISP2 allows *Leishmania spp.* to evade NET-mediated killing and persist within neutrophils [92].

Neutrophils influence infection trajectories through cytokine secretion [67, 93]. Infection induces IL-8 production, recruiting more neutrophils, and pro-inflammatory cytokines like IL-1β and TNF. [67]. Counterintuitively, *Leishmania spp.* simultaneously induces the production of transforming growth factor-beta (TGF-β) from neutrophils [66, 67, 81, 94–96]. TGF-β plays a pivotal role in suppressing IL-2 receptor (IL-2R) expression and IFN-γ-mediated responses, both of which are essential for effective anti-leishmanial immunity [97]. Moreover, TGF-β has been identified as a key determinant of host susceptibility to leishmania infection in vivo [97, 98].

In summary, recent evidence confirms a proparasitic role for neutrophils in the establishment of cutaneous infection; however, their overall role in *Leishmania* pathogenesis remains widely debated. Prophylactic depletion of neutrophils using Clone NIMP-R14 mAb 6 h before *L. major* infection led to reduced lesion development and parasite burden in BALB/c mice [99]. Interestingly, this protective effect was not observed in neutrophil-depleted C57BL/6 mice, highlighting a host-specific role for neutrophils [99]. Neutrophil depletion in mice has been associated with enhanced early parasite growth at the infection site and in the draining lymph nodes, yet a reduction in parasite burden is observed one to four weeks post-infection, as previously reviewed [100]. These findings suggest that neutrophils may facilitate parasite survival during early infection while contributing to parasite clearance at later stages. Consequently, neutrophils are critically involved in the establishment of CL, though their role in antileishmanial immunity appears paradoxical [66, 93]. Recent studies have proposed that neutrophils at the site of infection promote hypoxia and drive lesion development via CD8+ T cell-mediated mechanisms [101]. Additionally, Rosa-ERT2-CRE-induced depletion of Ly6G has demonstrated altered kinetics of *L. major* tissue entry without significantly impacting overall disease severity [102].

Further research into the interactions between *Leishmania* parasites and neutrophils, as well as the mechanisms by which the parasites subvert neutrophil defenses, is crucial. This knowledge is particularly important for developing effective prophylactic treatments, including vaccines. Much remains to be uncovered before a comprehensive understanding of the multifaceted and paradoxical role of neutrophils in leishmaniasis can be achieved.

## Macrophages

Macrophages are myeloid-derived phagocytes that play a critical role in the innate immune response [103, 104]. Like neutrophils, they contribute to host defense by clearing and recycling cellular debris through efferocytosis [105]. Unlike neutrophils, however, macrophages are primarily tissue-resident cells, participating in ongoing tissue surveillance and exhibiting long lifespans. Despite their essential role in immune defense, macrophages can be subverted by various pathogens, including *Leishmania* parasites, to establish intracellular infections [106–108].

As previously discussed, *Leishmania spp.* is transmitted to mammalian hosts through the bite of female phlebotomine sand flies [109]. Once internalized by phagocytes, the vector-borne metacyclic promastigotes transform into replication-competent amastigotes that are resistant to reactive oxygen species (ROS). This transformation enables their survival and proliferation within the hostile environment of the phagolysosome. From this intracellular niche, *Leishmania* parasites replicate until the host cell lyses, facilitating their dissemination unless immune intervention occurs [110]. *Leishmania* parasites have evolved to not only survive but also replicate and actively subvert macrophage killing mechanisms, underscoring the sophistication of their immune evasion strategies (Fig. 3).

Macrophages serve as the reservoirs—the major cell types within which *Leishmania* parasites hide, survive and proliferate to establish infection—and site of replication for *Leishmania spp. in vivo* [111]. As such, the interaction between the parasite and macrophages can significantly influence the subsequent development of antileishmanial immunity. *Leishmania spp.* predominantly enter macrophages through receptor-mediated phagocytosis [112, 113]. This process can result in either ‘silent entry,’ which allows the parasite to avoid immune detection, or ‘active entry,’ which stimulates an immune response [112, 113]. The outcome depends on the specific receptor engaged by the parasite and the *Leishmania spp.* involved, leading to highly variable immune responses [113]. Several receptor types have been implicated in the entry of *Leishmania* into macrophages, including complement receptors (CRs), Fc receptors (FcRs), and pattern recognition receptors (PRRs). These receptors play a crucial role in determining the nature of the immune response, influencing whether the parasite evades detection or triggers an inflammatory response.

‘Silent entry’ into macrophages via complement receptor 3 (CR3; CD11b/CD18) has long been considered an important mechanism of uptake in leishmaniasis [112, 113]. By exploiting opsonization with inactivated C3b (iC3b), *Leishmania* spp. can enter phagocytes without triggering the parasite-killing mechanisms [78]. CR1, which recognizes C3b, has been shown to assist in CR3-mediated uptake but cannot compensate for CR3 in its absence [114, 115]. Engagement of CR3 has also been demonstrated to inhibit IL-12 production and macrophage activation [116–118]. However, studies show that CD11b deficiency results in only a modest reduction in *L. major* infection severity in susceptible BALB/c mice, and no difference in resistant C57BL/6 mice [118]. These findings suggest that CR3 may not play a major role in *L. major* uptake, indicating species-specific differences [119]. More recent studies have shown that when infectious *L. infantum* metacyclic promastigotes engage CR3, they induce ‘actin-rich ruffles’ that seem to facilitate parasite uptake and survival. Interestingly, CD11b-deficient macrophages did not show impaired phagocytosis in this context [120].

Since *L. donovani* and *L. infantum* are typically associated with VL, this suggests potentially differential roles for CR3 in mediating visceral versus cutaneous *Leishmania* infections, although this hypothesis requires further investigation. Notably, the study did demonstrate a significant increase in LAMP1 positivity, which is indicative of phagosome maturation [120]. In line with this, studies utilizing bone marrow-derived macrophages (BMDM) deficient in CD11b show significant delays in phagosome maturation following *L. donovani* infection [121]. Taken together, these findings suggest that parasites opsonized with iC3b and internalized via CR3 may influence the maturation of the phagolysosome, thereby affecting parasite killing.

Phagosome maturation is closely linked to the ability of the parasite to persist within the phagolysosome. In a *L. donovani* infection model, comparing *FcγR*^−/−^ and *Cd11b*^−/−^ BMDM to wildtype BMDM, *FcγR*^−/−^ and *Cd11b*^−/−^ BMDM exhibited significant decreases in LAMP1 positivity at early timepoints [122]. FcγR, which is cognate for IgG, suggests that opsonization with antibodies—beyond complement—also influences phagosome maturation. In addition to complement receptors, research has highlighted the role of other receptor interactions, both with and independent of CR3. Toll-like receptors (TLRs), key pattern recognition receptors (PRRs), are implicated in macrophage entry and the subversion of host defenses [112, 123]. Specifically, TLR2 has been shown to impact phagosome maturation, which, as previously mentioned, can lead to impaired parasite killing [122]. TLRs recognize microbial pathogens via pathogen-associated molecular patterns (PAMPs) [124]. The agonization of TLRs by *Leishmania* spp. varies depending on the surface expression of virulence factors, which differ across species. Emerging vaccine strategies aim to exploit TLR agonists as adjuvants to generate effective Th1-based immunity against *Leishmania* spp. [125, 126]*.* Among these, agonists targeting TLR2 have drawn particular interest. However, the role of TLR2 in leishmaniasis remains controversial and unclear [125–129]. TLR2 has been shown to have both anti-parasitic and pro-parasitic effects, depending on the species [130].

LPG, a highly expressed virulence factor in *Leishmania spp.*, can agonize TLR2 following infections with *L. major*, *L. mexicana*, *L. aethiopica*, and *L. tropica*, though the strength of this interaction varies [130, 131]. In macrophages, LPG has been shown to induce TLR2-dependent reactive oxygen species (ROS) production following *L. major* infection [130, 132]. Another study reported that *L. major* or *L. mexicana* infection in TLR2-deficient mice on a resistant background led to significantly larger lesions, which the authors attributed to the promotion of Th2 immunity, with elevated IL-4, IL-13, and IL-10 levels [133]. Additionally, using *lpg1−/− L. mexicana*, the study demonstrated that the LPG-TLR2 interaction was not required to mediate this phenotype.

The PRR mannose receptor (MR; CD206) has been shown to bind *Leishmania* spp., but its role in parasite uptake and disease outcome, like the other receptors discussed, remains controversial [134, 135]. It was initially hypothesized that MR recognition of highly glycosylated surface molecules on *Leishmania spp.*, including the major virulence factor LPG, could influence phagocytosis and the immune recognition of the parasite [123, 136]. Early *in vitro* studies suggested that MR might play a role in adhesion and phagocytosis of *L. donovani*, but subsequent research has shown that infection of MR-deficient mice with *L. major* or *L. donovani* resulted in clinical outcomes similar to those of wild-type controls [123, 135, 137]. *In vitro* studies with MR-deficient BMDM also showed normal production of cytokines (TNF and IL-12), activation of MAPK signaling, and phagocytosis of *L. major* and *L. donovani*, comparable to control macrophages [137]. However, a more recent study suggested that MR plays a specific role in non-healing cutaneous *L. major* (*LmSd*) infections within a Th1 environment [68]. This study demonstrated stronger M2 polarization following IL-4/IL-10 stimulation, showing that these cytokines are essential for maintaining the MR^hi^ dermal macrophage population [55]. These findings suggest that the impact of MR could be tissue-specific, and that MR recognition of *Leishmania* may promote parasitic survival, though the conflicting data warrants further investigation. It is also possible that MR expression on other phagocytic cells could be more relevant than its expression on macrophages. A clearer understanding of MR’s role during leishmaniasis will be crucial for evaluating the efficacy of emerging treatments [138–140]. Additionally, MR is a key marker of M2 polarization, which critically affects the macrophage’s ability to kill parasites.

Due to their role as a reservoir and site of replication, the ability of macrophages to kill *Leishmania* parasites is critical for controlling the infection. Macrophage killing of *Leishmania* parasites is highly dependent on their activation phenotype. Classically activated (M1) macrophages can efficiently kill amastigotes within the phagolysosome through the production of nitric oxide (NO) [141]. NO production is mediated by the enzyme inducible nitric oxide synthase (iNOS), and iNOS-mediated NO production has been shown to be crucial for clearing *Leishmania* infection in mice. iNOS deficiency impairs the ability to cleave the amino acid L-arginine into NO, thereby hindering the macrophage’s ability to penetrate the phagolysosome and kill amastigotes. The activation of iNOS is upstreamed by JAK/STAT signaling, which is critically induced by IFN-γ binding to the IFN-γ receptor (IFNGR) on macrophages during *Leishmania spp.* infection [142]. Extensive evidence has shown the importance of IFN-γ production by CD4+ Th1 cells, which is crucial for macrophage activation [143]. The balance between classical and alternative macrophage activation is largely governed by cytokines, with IFN-γ promoting classical activation (M1), and cytokines such as IL-4, IL-10, and TGF-β favoring alternative activation (M2).

While these cytokines can be produced by various immune cells, including NK cells and CD8+ T cells, it is the cytokine production by CD4+ T cells that primarily drives the immune response. A response favoring Th1 and Th1-associated cytokines is strongly correlated with better disease outcomes, whereas a Th2-skewed response is associated with higher parasite burdens and worse disease outcomes. This has been extensively reviewed in the literature and will be discussed in more detail in the dendritic cell and T cell sections of this review.

## Natural killer cells

**Figure 4:**
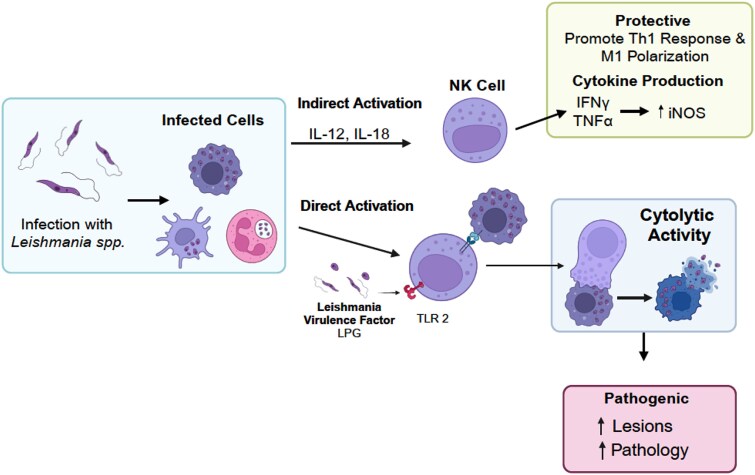
Protective and pathogenic role of NK cells during *Leishmania* infection: NK cells activation is linked to both protective and pathogenic outcomes in leishmaniasis. Direct activation is correlated with increased lesion size and pathology, while indirect activation is correlated with protective cytokine production.

Natural Killer (NK) cells are granulocytic lymphocytes that recognize intracellular infection and cancerous cells, initiating cytotoxic responses against them [144]. There is a growing interest in utilizing NK cell-based immunotherapies for the treatment of leishmaniasis, highlighting the need for a deeper understanding of NK cell function and their potential as vaccine targets against *Leishmania spp.* [145]. Interestingly, suppression of NK cell activity has been reported in response to *L. major* infection, with the virulence factor gp63 directly inhibiting NK cell proliferation [146]. Additionally, human-derived NK cells upregulate the activation marker CD69 upon exposure to *Leishmania* promastigotes, though the extent of activation varies depending on the *Leishmania* species. The parasites ability to inhibit NK cell proliferation, combined with NK cells’ production of protective cytokines such as IFN-γ and TNF, suggests that NK cells play a significant role in *Leishmania* clearance [147]. However, the role of NK cell in *Leishmania* infection remain complex and poorly understood, with studies reporting both protective and pathogenic functions [148–150]. Further research is needed to fully elucidate their contribution to host immunity and pathogenesis in *Leishmania* infections (Fig. 4).

Although NK cells perform cytolytic functions similar to CD8 T cells, they do not require antigen-specific recognition to exert their cytotoxic effects [151, 152]. Notably, *Leishmania*-infected macrophages do not appear to be primary targets of NK cell-mediated cytolysis *in vitro* and *in vivo* [147, 153]. One study demonstrated that in patients with *L. braziliensis*-mediated CL, NK cells produced higher levels of IFN-γ, TNF, perforin, and granzyme B compared to CD8 T cells, relative to healthy controls [150]. Additionally, NK cell-derived granzyme B has been implicated in promoting inflammation.

Conversely, NK cell-derived cytokines have been associated with both protective and detrimental infection outcomes. NK cells serve as a significant source of IFN-γ, which promotes Th1 differentiation and enhances resistance to *L. major* infection [147]. More specifically, NK cell secretion of IFN-γ and TNF has been correlated with increased inducible nitric oxide synthase (iNOS) expression [153]. As previously mentioned, iNOS plays a critical role in macrophage-mediated production of nitric oxide (NO), which is essential for eliminating intracellular *Leishmania* parasites [154]. Additionally, NK cell-induced maturation and activation of dendritic cells is associated with enhanced IFN-γ and TNF production [155, 156]. While interactions between NK cells and DCs have been shown to amplify inflammation in various contexts, their role in *Leishmania* infection remains largely unexplored [148, 157]. IL-18 and TLR9-induced IL-12 have been implicated in NK cell activation in experimental *L. infantum* infection, whereas IL-5 has been linked to NK cell proliferation [144, 158]. Notably, IL-18 deficiency results in a significant reduction in IFN-γ production, cytotoxic activity, and FasL expression [144]. Furthermore, NK cell-derived granzyme B has been observed in *L. braziliensis*-mediated CL [150].

While cytokines can indirectly activate NK cells, they can also be directly activated through engagement of TLR2 [159]. The direct activation of NK cells via TLR2 engagement has been inversely correlated with diffused CL (DCL), highlighting a potential protective role for these cells [160]. The *Leishmania* virulence factors LPG has been reported to activate human NK cells through TLR2 signaling [161]. Notably, NK cell MFI of TLR1, TLR2, and TLR6 was decreased in DCL, both with and without LPG stimulation [160]. NK cells from DCL patients exhibited decreased IFN-γ production, as demonstrated by qRT-PCR analysis of peripheral blood cells and lesion staining. Interestingly, these NK cell defects were not observed in NK cells from localized CL (LCL) patients. Furthermore, the frequency of NK cells in the peripheral blood and lesions of LCL patients were significantly higher compared to those with DCL [160]. Collectively, these findings suggest that NK cell cytokine production following TLR engagement may play a crucial role in preventing disease progression from LCL to DCL.

## Dendritic cells

Dendritic cells (DCs) comprise a highly heterogeneous population of myeloid cells. DCs are renowned for their ability to efficiently present antigens, which is essential for priming effective, antigen-specific T cell responses. Several subsets of DCs, each with distinct functions and phenotypic markers, derive from common DC progenitors. Classical dendritic cells (cDCs) are professional antigen-presenting cells and can be further divided into two main subtypes. cDC1s are primarily known for their role in activating CD8+ T cells and cross-presenting antigens, while cDC2s (CD11b+; SIRPα/CD172a+) are key in CD4+ T cell polarization [16]. cDC1s can be further subdivided into CD103+ or CD8α + populations based on functional differences [162]. Plasmacytoid dendritic cells (pDCs) can also serve as APCs but are best known for their ability to produce large amounts of type I interferons (IFN-I) [163, 164]. Additionally, monocytes can differentiate into a subset of inflammatory monocyte-derived DCs (MoDCs) [165].

**Figure 5:**
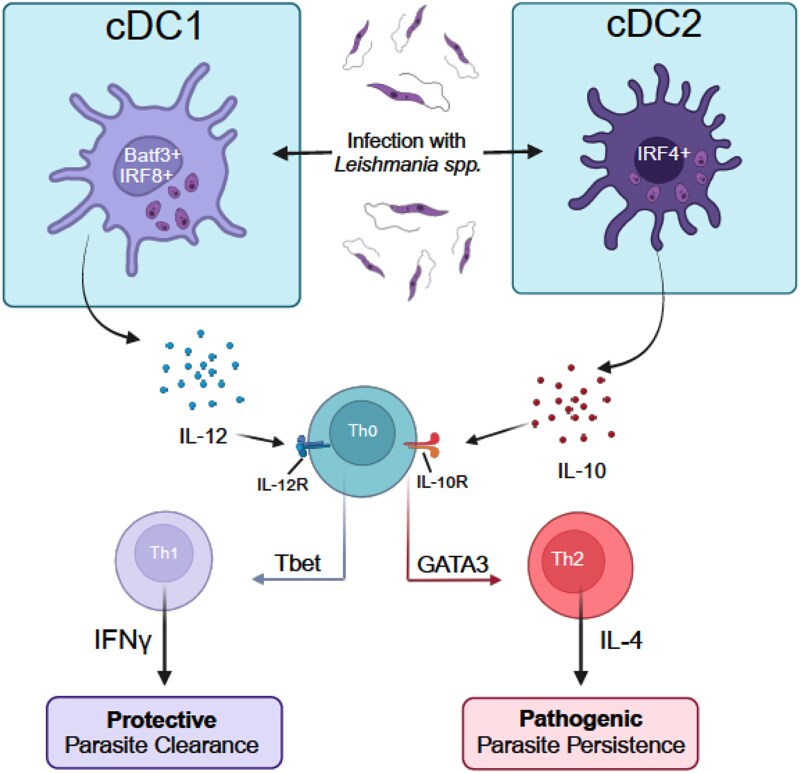
Dendritic cells and leishmaniases. Dendritic cells are critical determinants of CD4+ T cell polarization, and as such are critical for determining disease outcomes in leishmaniases. cDC1s: cDC1s are responsible for most of the IL-12 production following *Leishmania spp.* infection, and sustained IL-12 production is required to develop and maintain a protective Th1 dominant environment. cDC2s: While the role of cDC2s is not as well understood, there is sufficient evidence to suggest that their production of IL-10 prevents effective establishment of Th1 response and therefore can hinder antileishmanial immunity.

Like macrophages, DCs can also phagocytose *Leishmania* parasites. However, unlike macrophages, DCs do not serve as major sites of parasite replication or direct killing. This may be due to the shorter lifespan of DCs, which limits their capacity to support parasite survival. As a result, *Leishmania* parasites likely develop strategies to enhance their survival and subvert immune responses within macrophages. Despite this, DCs, with their unmatched ability to present antigens to T cells, are pivotal for developing antileishmanial immunity (Fig. 5). Specifically, DCs are critical drivers of antileishmanial Th1 immunity through the production of IL-12 [21, 166–168]. Additionally, certain DC populations can promote Th2 polarization, which contributes to increased susceptibility to disease [140, 169–171].

Infection of dendritic cells (DCs) with *L. major* results in functional IL-12 production, which is dependent on CD40-CD40L interactions [166]. In situ, DCs, but not macrophages, produce IL-12 immediately upon infection with *L. donovani* [167]. However, a study of human DCs showed that *L. donovani* infection does not prime these cells to produce IL-12, suggesting potential species-specific differences between mice and humans [172]. Most importantly, sustained IL-12 production is required for Th1 development and is crucial for maintaining Th1 responses following *L. major* infection [53, 54, 173]. Genetically resistant mice deficient in IL-12 are susceptible to *L. major* infection and exhibit a Th2 bias like that of susceptible BALB/c mice [174]. IL-12/IL-4 double knockout mice infected with *L. major* confirm that the requirement for IL-12 is not due to the inhibition of IL-4-mediated Th2 immunity; IL-12 is still necessary in the absence of a Th2 response to generate a Th1 response [53]. Taken together, these findings suggest that DCs are a major source of IL-12 and are essential for driving Th1 polarization during *Leishmania* spp. infection [21, 53].

However, as previously reviewed, the ability of different DC subsets to produce IL-12 appears to be inversely correlated with their susceptibility to infection [162, 171, 175]. The ability of DCs to promote Th1 or Th2 polarization—via IL-12 or IL-10 production, respectively—seems to be subset-dependent. cDC1s generally promote Th1 polarization, while cDC2s tend to promote Th2 polarization [175]. This is further supported by studies focusing on critical transcription factors that differentiate cDC1 and cDC2 subsets. For instance, Batf3, along with IRF8., are specific to cDC1s, while IRF4 and KLF4 are specific to cDC2s [176, 177].

Batf3, or basic leucine zipper transcription factor ATF-like 3, is a critical transcription factor in the development and function of cDC1s [178–180]. Mice deficient in Batf3 lack all cDC1 cells [180, 181]. Both genetically susceptible and resistant Batf3-deficient mice develop exacerbated and unresolving cutaneous pathology following even a low-dose infection with *L. major* [168, 181]. Adoptive transfer of wild-type (WT), but not *Il12p40*-deficient DCs, significantly reduces the pathology in *Batf3*^−/−^ mice [181]. This data indicates that Batf3 is required by CD103+ DCs to produce adequate IL-12 following *L. major* infection. While Batf3 deficiency does not prevent the presentation of *L. major* to T cells, the differentiation of the T cells becomes skewed towards Treg and Th2 phenotypes. Further studies are needed to explore these findings in more detail.

A critical role for Batf3 in IL-12-mediated antileishmanial immunity was also validated in *L. infantum*-infected mice, where Batf3-deficient mice failed to control hepatic parasitosis [182]. This suggests an essential, though indirect, role for cDC1s in parasite control via IL-12 production. Furthermore, another study of *L. major* infection in *Batf3*-deficient mice showed that DCs harvested from the draining lymph node (dLN) of *Batf3*-deficient mice produced less IFN-γ. In contrast, CD8α + cDC1s from the dLN of *L. major*-infected mice induced more IFN-γ production by T cells than CD103+ cDC1s [168]. Together, this data underscores that the transcription factor Batf3, and by extension cDC1s, are crucial for inducing adequate IL-12 production by dendritic cells and for subsequent antileishmanial immunity in experimental models of both CL and VL. The ability of cDCs to induce protection following infection with *Leishmania* spp. appears to be highly temporal. For example, DTR mice depleted of Batf3 or WT mice depleted of ‘cross-presenting DCs’ between days 17–19 post-infection showed significantly larger lesions following *L. major* infection [168, 183].

In addition to Batf3, interferon regulatory factor 8 (IRF8) has recently been identified as a critical transcription factor for the differentiation of DC precursors into cDC1s [176, 177]. IRF8 deficiency results in the reprogramming of cDC1s into cDC2s [184]. Both IRF8 and IRF1, which are upstream of IL-12, are upregulated in human DCs following *L. major* infection. Notably, NF-κB was reported to be upregulated alongside IRF1 and IRF8 [172]. Interestingly, this study also showed that prophylactic inhibition of NF-κB blocked *L. major*-mediated induction of IRF8, IRF1, and IL-12 in human DCs. Whether this signaling cascade is induced directly or indirectly by *L. major* remains unclear.

Current evidence suggests that IRF8-mediated immunity to intraphagosomal pathogens, such as *Leishmania* spp., is regulated by natural resistance-associated macrophages (Nramp1) [185]. In dendritic cells, Nramp1 (also known as Slc11a1) is known to influence MHCII expression and APC function [186]. One study demonstrated that Nramp1 colocalizes with LAMP1+ vesicles in DCs, where *Leishmania* parasites reside during infection [186, 187]. Additionally, a meta-analysis revealed that multiple polymorphisms in the SLC11A1 gene are associated with increased susceptibility to either CL or VL in affected individuals [188]. Further studies are needed to clarify the roles of IRF8 and Nramp1 expression by DCs in IL-12 production and corresponding susceptibility. These studies may provide a deeper mechanistic understanding of cDC1-mediated immunity to *Leishmania* spp. and, by extension, cDC2-mediated pathology.

While Batf3 and IRF8 are known to promote IL-12, IRF4 has been shown to inhibit IL-12 production by DCs following *L. major* infection [171]. Previous reports also demonstrate that in DCs, IRF4 can promote Th2 differentiation [169, 170]. IRF4 is largely restricted to cDC2s. Examining how IRF4 expression by DCs influences disease outcomes could reveal more specific functions of cDC2s following *Leishmania* infection.

IL-4 production has been linked to the progression of infections caused by multiple *Leishmania spp.* [189]. In BALB/c mice, a large burst of IL-4 is attributed to their genetic bias toward Th2 polarization and subsequent susceptibility. Counterintuitively, IL-4Rα ablation in CD11c + DCs further exacerbated disease in BALB/c mice infected with *L. major* [190]. Another study using this model showed that IL-4 responsiveness in dendritic cells is dispensable in the context of *L. mexicana* infection [191]. Together, these findings suggest that species-specific differences may determine the necessity of IL-4Rα signaling on dendritic cells. A recent report has now demonstrated that IL-4Rα signaling regulates inflammatory MoDCs, which are required for the control of *L. major* infection [192].

## T cells

T cells play a pivotal role in the adaptive immune system, primarily contributing to cell-mediated immune responses within the host [193]. They can be broadly categorized into two major groups: T helper cells that express CD4 markers (historically known as L3T4), and Cytotoxic T cells that express CD8 markers (historically known as LYT2 and LYT3). These are now commonly referred to as CD4+ and CD8+ T cells, respectively. CD4+ T cells function by assisting B cells and producing specific cytokines, while CD8+ T cells produce pro-inflammatory cytokines such as IFN-γ and TNF and directly kill infected target cells.

CD4+ T cell differentiation depends on various factors, including endogenous cytokines and transcription factors [194–197]. CD4+ T cells can be further classified into five major subtypes based on their signature cytokines and lineage-specific master transcription factors [198–200]:

***T helper 1 (Th1)*:** Cytokine IFN-γ and transcription factor T-bet.

***T helper 2 (Th2)*:** Cytokine IL-4, IL-5 and IL-13, and transcription factor GATA3.

***T helper 17 (Th17)*:** Cytokine IL-17 and IL-22 and transcription factor RORγt.

***T follicular helper (Tfh)*:** Cytokine IL-21 and transcription factor Bcl6.

***T regulatory (Treg)*:** Cytokine IL-10, TGF-β and IL-35, and transcription factor FOXP3.

**Figure 6:**
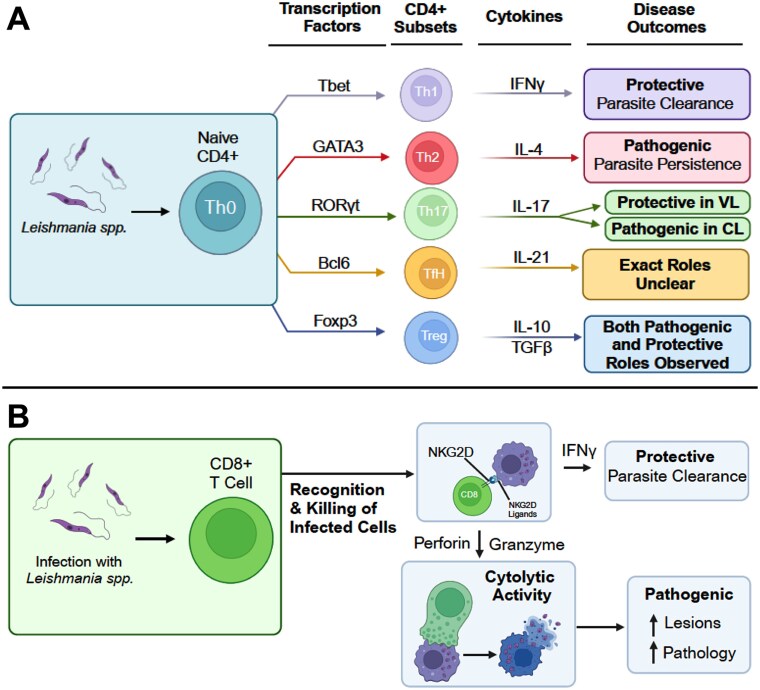
T cells in leishmaniasis. T cells, broadly subdivided into CD4+ and CD8+ T cells, play central roles in regulating adaptive immune responses during Leishmania spp. infections. (A) CD4+ T cells: Naive CD4+ T cells that encounter *Leishmania* spp. in the context of specific cytokines will adopt a specific T helper (Th) phenotype and subsequently influence disease outcome. CD4+ T cell subsets and their proposed roles in regulating *Leishmania spp.* infections. (B) CD8+ T cells: Naive CD8+ T cells once activated can have both protective and pathogenic role during *Leishmania spp.* infections.

In this review, we will examine and discuss the roles and function of these specific cell subtypes during *Leishmania* spp. infection (Fig. 6).

## T helper 1 (Th1) cells

Numerous studies have demonstrated that the outcome of infections often depends on which CD4+ T cell subset is activated. T helper 1 (Th1) and T helper 2 (Th2) cells are distinct subsets of CD4+ T cells that mediate well-defined immune responses. Th1 cells are primarily involved in cell-mediated immunity, while Th2 cells drive humoral immunity [195]. Specifically, during *Leishmania spp*. infections, Th1 cells are associated with resistance to infections, while Th2 cells are linked to susceptibility [194, 195]. Th1 cells are characterized by the production of IL-2 and IFN-γ in response to antigens, whereas Th2 cells produce IL-4, IL-5, and IL-13 [194, 198, 201–207]. T-bet, a transcription factor specific to Th1 cells, plays a crucial role in Th1 development by inducing IFN-γ production [208]. In contrast, transcription factor GATA3 and IL-4 are essential for Th2 cell differentiation [194, 195].

In the context of *Leishmania* spp. infections, resistance or susceptibility in mice is predominantly influenced by Th1 or Th2 polarization and their associated cytokines [194, 209]. *Leishmania*-susceptible BALB/c mice exhibit a strong Th2 response, characterized by high levels of IL-4, while *Leishmania*-resistant C57BL/6 mice display a robust Th1 response, marked by IFN-γ production.

Several inbred mouse strains, such as 129/Sv/E, C57BL/6, and C3H, are resistant to *L. major* infection, a resistance that is directly attributed to their robust Th1-type immune responses. [174, 194, 210, 211]. Th1-associated IFN-γ cytokines directly promote macrophage activation and facilitate the intracellular killing of parasites within macrophages [212–215]. Lymph nodes from *L. major*-infected 129/Sv/E mice, when stimulated with *Leishmania* antigens, produce high levels of IFN-γ [174]. IFN-γ receptor (IFNGR) knockout 129/Sv/E mice showed severe disease progression compared to wild type mice [211]. Similarly, treating wild-type resistant 129/Sv/E mice with anti-IFN-γ renders them susceptible to *L. major* infection [211]. While C57BL/10 mice infected with *L. major* typically heal, infection with *L. amazonensis* is prolonged and non-healing [216]. The healing phenotype in *L. major*-infected mice is associated with higher IFN-γ production and Th1 differentiation compared to *L. amazonensis*-infected mice [216].

IL-12, a cytokine secreted by dendritic cells, is essential for both the development and maintenance of Th1-type responses during *Leishmania spp.* infection [54]. IL-12-deficient 129/Sv/E mice develop severe disease following *L. major* infection, accompanied by reduced IFN-γ production, highlighting the protective role of IL-12 in resistant mice [174]. In susceptible BALB/c mice, treatment with recombinant murine IL-12 promotes disease resolution by enhancing IFN-γ production and reducing IL-4 levels [217]. This underscores the role of IL-12 in Th1 differentiation and resistance to *Leishmania spp.* infection [217].

T-bet, a transcription factor critical for Th1 differentiation, regulates IFN-γ production [208]. T-bet-knockout C57BL/6 mice infected with *L. major* exhibit increased susceptibility compared to wild-type C57BL/6 mice, correlating with reduced parasite-specific T cell IFN-γ production *in vitro* [218]. In non-rodent hosts, *L. infantum* infection has been reported to induce high levels of Th1 cytokines, such as IFN-γ in crossbreed dogs and IL-2, in Cirneco dell’Etna dogs [219]. The cytokines involved in Th1 responses and their association with resistance to *Leishmania spp.* infections have been extensively reviewed [220].

The coexistence of *Leishmania*-reactive T cells producing both Th1 and Th2 cytokines has been reported, with cytokine presence detectable years after recovery from infection [221, 222]. The diverse types of self-limiting and severe leishmaniasis in humans, along with their associated T helper cell responses, have been extensively reviewed [223]. *In vitro* experiments demonstrate that PBMCs from active and recovered *Leishmania* patients produce high levels of IFN-γ when stimulated with soluble *Leishmania* antigens. However, PBMCs from non-healing patients exhibit little to no IFN-γ production [224]. In humans, localized cutaneous leishmaniasis caused by *L. braziliensis* is characterized by the predominance of type 1 cytokine mRNAs, such as IL-2 and IFN-γ, within lesions [225]. Among cutaneous leishmaniasis cases, peripheral blood mononuclear cells (PBMCs) from patients with mild disease produce higher levels of IFN-γ compared to those with severe disease, highlighting the association between Th1 responses and disease severity [226]. Another study observed the intralesional expression of IL-10 and IL-12 in patients with active localized cutaneous leishmaniasis, suggesting that IL-10 may act as a regulatory mechanism to control IL-12-induced Th1 responses [227]. Altogether, these studies demonstrate the role of Th1 cells and IFN-γ in providing protection during *Leishmania spp*. infections.

## T helper 2 (Th2) cells

Th2 cells are a subset of CD4+ T cells that function as helper cells to augment humoral immunity by promoting antibody production. However, they also inhibit certain cell-mediated immune responses, which can increase susceptibility to infections [198, 225]. Consequently, Th2 cells are often associated with severe disease or heightened susceptibility to infections [228]. Th2 cells are primarily characterized by their production of cytokines such as IL-4 and IL-5 [194, 195, 201–203]. Notably, during *Leishmania* spp. infection, the production of IL-4 and IL-5 is specific to CD4+ T cells and not CD8+ T cells [229]. Although IL-4 is widely regarded as a key driver of Th2 differentiation [194, 195], its role in *Leishmania* spp. infections remains ambiguous [230].

Studies in murine models have provided insights into the role of Th2 cells in *Leishmania* spp. infections. For instance, *L. major* infection in susceptible BALB/c mice is associated with a predominant Th2-type response, leading to non-healing disease [204]. Lymphoid tissues from *Leishmania*-infected BALB/c mice exhibit IL-4 mRNA expression, further implicating Th2 cells in disease progression [210]. Deletion of CD4+ T cells using the GK1.5 anti-CD4 antibody prior to infection induces a healer phenotype in BALB/c mice, marked by decreased IL-4 expression and increased IFN-γ production [210]. Additionally, neutralizing IL-4 with antibodies in *Leishmania*-infected BALB/c mice significantly attenuates disease progression [210, 230, 231]. These findings underscore the role of Th2-specific IL-4 in both disease progression and Th2 differentiation. Interestingly, BALB/c mice treated with IL-4 neutralizing antibodies not only recover from the disease but also exhibit protection against reinfection [232].

Another study demonstrated that a balance exists between Th1 and Th2 cells in *L. major*-infected BALB/c mice. It was hypothesized that antigen-presenting cells preferentially activate Th2 cells during infection, tipping the balance in favor of susceptibility [233]. Furthermore, transgenic mice engineered to produce IL-4 on a resistant genetic background were rendered susceptible to *Leishmania spp.*, despite unchanged or reduced IFN-γ levels in these mice [234, 235]. In another model, severe combined immunodeficient (SCID) mice lacking mature B and T lymphocytes developed severe disease when transferred with Th2- CD4+ cells producing IL-4 and IL-5 from *L. major*-infected mice, as compared to non-transferred SCID mice [236]. These data suggest that the severity associated with Th2 responses is transferable via either Th2-specific cytokines or cytokine-producing cells, even in a resistant environment.

Although IL-4 has been implicated in Th2-mediated susceptibility, studies using genetic models with mice deficient in IL-4 have produced contradictory findings. For example, IL-4-deficient BALB/c mice exhibit both susceptibility [237] and resistance [238] to *L. major* infection, depending on experimental conditions. Importantly, acute IL-4 produced during the first few hours of *Leishmania spp.* infection were shown to instruct DC to produce IL-12, which helped generate Th1 responses [230, 239]. These findings highlight the complexity of IL-4’s role in *Leishmania* spp. infections and suggest that the contribution of IL-4-producing Th2 cells to susceptibility requires further evaluation, specifically in the context of IL-4 KO genetic models.

Th2 cells are also associated with anti-inflammatory properties [240]. In macrophages, the enzymes iNOS converts L-arginine into NO, whereas Arginase-1 converts L-arginine into ornithine [241]. NO production is typically linked to M1 macrophages and is associated with antimicrobial and anti-proliferative responses, whereas ornithine production is characteristic of M2 macrophages and is involved in cell proliferation, tissue repair, and wound healing [241]. Previous studies have demonstrated that Th2 cells and Th2-associated cytokines, such as IL-4 and IL-10, selectively induce Arginase-1 but not iNOS in murine bone marrow-derived macrophages and dendritic cells *in vitro* [240]. In *L. major*-infected BALB/c and C57BL/6 mice, Arginase-1 expression correlates with footpad swelling, suggesting its involvement in disease pathology. Moreover, in susceptible BALB/c mice, pharmacological inhibition of Arginase-1 was found to delay disease progression [242]. Collectively, these findings highlight the role of Th2 cells in establishing an anti-inflammatory or tissue-repair environment that facilitates parasite persistence and contributes to disease progression.

## T helper 17 (Th17) cells

Th17 cells represent a distinct subpopulation of CD4+ T cells characterized by the production of IL-17 cytokines and transcription factor RORγt [243–249]. These cells also produce other cytokines, including IL-17A, IL-17F, IL-21, IL-22, TNF, IL-6, and IL-26 [246, 247, 250]. Initially identified as pathogenic due to their involvement in autoimmune diseases such as EAE and colitis, their roles in infectious and non-infectious settings are being actively studied [243, 251–254]. This section reviews the involvement of Th17 cells in *Leishmania* spp. infections.

The role of Th17 during *Leishmania spp.* infection can be protective or pathogenic depending on the type of leishmaniasis and host [255, 256]. Significant roles for Th17 cells have been reported in post-kala-azar dermal leishmaniasis (PKDL), a dermal sequel of visceral leishmaniasis (VL) [257]. In PKDL patients, IL-17 gene expression and IL-17-positive cells were found to be significantly higher in tissue lesions compared to controls. Similarly, other Th17-related cytokines, including IL-1β, TGF-β, and IL-6, were also upregulated in PKDL. Notably, IL-6, IL-23, and IL-1β are essential for the induction and maintenance of the Th17 response [249, 257, 258]. Peripheral blood mononuclear cells (PBMCs) from PKDL patients produced elevated levels of IL-17 and IL-23 upon exposure to *Leishmania* antigens [257]. A study by Pitta et al. (2009) demonstrated that *L. donovani* strongly induces IL-17 and IL-22 in PBMCs from healthy individuals [255]. Furthermore, PBMCs exposed to *L. donovani* antigens from VL-resistant individuals produced significantly higher levels of IL-17 and IL-22 compared to the disease group, suggesting a protective role for Th17 cells [255]. Additional studies confirmed the protective role of Th17 cells in VL, showing reduced *L. donovani* burdens in the spleens and livers of BALB/c mice treated with recombinant IL-23 and IL-17 [259]. Asad et al. demonstrated that *L. donovani* infection in susceptible BALB/c mice resulted in the accumulation of splenic Th17 cells and IL-17 cytokines in both the spleen and serum [260]. Notably, serum IL-17 levels inversely correlated with liver parasite burden, supporting a protective role for Th17 cells [260]. Another study using *Il17ra*-deficient C57BL/6 mice during *L. infantum* infection found increased susceptibility, suggesting IL-17A acts synergistically with IFN-γ to aid parasite killing [261]. Together, these studies highlight the protective role of Th17 cells in VL in both humans and susceptible BALB/c mice.

In contrast, certain findings suggest a pathogenic role for Th17 cells during *Leishmania spp.* infections. For instance, a 2016 study demonstrated that IL-17A knockout (KO) C57BL/6 mice were highly resistant to VL following *L. donovani* infection [39]. These mice exhibited significantly reduced parasite burdens in the liver and spleen compared to wild-type mice, indicating that Th17 cells might exacerbate VL in C57BL/6 mice. Cellular analysis revealed that γδ + T cells were the predominant IL-17 producers, followed by CD4+ T cells, suggesting other immune cells could contribute to the observed phenotypes in IL-17A KO mice [39].

Studies comparing BALB/c and C57BL/6 mice have shown that the former, which are susceptible to *L. major*, produce higher levels of IL-17 and develop non-healing lesions, while the latter are resistant [256]. IL-17A KO mice on the BALB/c background showed reduced lesion development and lower *L. major* burdens compared to wild-type counterparts, highlighting a pathogenic role for IL-17 in susceptible BALB/c mice [256]. Interestingly, *in vitro* analyses revealed that in BALB/c mice, IL-17 is produced by CD4+ T cells, neutrophils, and to a lesser extent, γδ + T cells, suggesting non-Th17 sources of IL-17 during *Leishmania* spp. infections [256].

Additional studies from the Stebut research group further confirmed these findings, demonstrating that IL-17 and IL-23 (a key cytokine responsible for Th17 cell expansion) promote CL pathogenesis in BALB/c mice [262, 263]. Notably, IL-17 and IL-23p19 KO mice in the BALB/c background exhibited improved outcomes, including reduced parasite burden and faster lesion healing, compared to wild-type counterparts [262, 263]. Conversely, in C57BL/6 mice, IL-17 and IL-23 appeared less critical, as KO groups did not exhibit significant differences in healing times or parasite burdens [262, 263]. Kostka et al. also observed that IL-17 cytokines are primarily detected in the lesions of susceptible BALB/c mice, with negligible levels in C57BL/6 mice, explaining why IL-17 deficiency models are protective only in BALB/c mice [256]. Administration of recombinant IL-17A in C57BL/6 mice during *L. major* infection increased lesion size and parasite burden, further supporting a pathogenic role for IL-17 [256]. Similarly, overexpression of IL-17A in T cells in C57BL/6 mice resulted in worsened disease outcomes [263].

In human nonulcerated cutaneous leishmaniasis (NUCL) caused by *L. infantum chagasi*, skin lesions displayed high cellular densities of CD4+ T lymphocytes, RORγt +, IL-17+, TGF-β+, IL-6+, and IL-23+ cells [264]. Correlation analyses revealed strong relationships between IL-17+ and IL-23+ cells, as well as between RORγt + and CD4+ T cells, indicating the presence of Th17 cells and their involvement in inflammation [264]. While these findings suggest a pro-inflammatory role for Th17 cells in NUCL, further studies are required to confirm this hypothesis.

Taken together, these findings highlight the context-dependent roles of Th17 cells in *Leishmania spp.* infections. Evidence from VL suggests a predominantly protective role for Th17 cells in humans and experimental models, particularly in susceptible hosts like BALB/c mice. However, genetic validation in susceptible hosts remains limited and will need further validation. In contrast, Th17 cells play a pathogenic role in CL, as demonstrated by various genetic models and experimental studies. These findings underscore the dual nature of Th17-mediated immunity, which can be either protective or pathogenic, depending on the clinical manifestation (VL or CL) and the host’s genetic background.

## T follicular helper (Tfh) cells

T follicular helper (Tfh) cells are a subset of CD4+ T cells characterized by the expression of the chemokine receptor CXCR5, IL-21, and PD-1 [265–268]. These cells are defined by the transcription factor Bcl6, which is both necessary and sufficient for Tfh differentiation [269–272]. Functionally, Tfh cells are crucial for providing help to B cells and for the generation of functional germinal centers (GCs).

Tfh cells have not been extensively studied in the context of *Leishmania* spp. infection. However, a study on visceral leishmaniasis (VL) following *L. infantum* infection in rhesus macaques (non-human primates) demonstrated an acute increase in splenic Tfh cells (defined by CXCR5, Bcl6, and IL-21 expression) around day 28 post-infection [273]. Immunofluorescence studies of splenic tissues revealed that these Tfh cells localized near B cells within germinal centers, and their increase positively correlated with memory B cell populations and antigen-specific antibody responses [273]. These findings align with earlier reports suggesting that Tfh cells located at the interface of the T cell zone and B cell follicle are critical for initiating extrafollicular antibody responses [274].

In a mouse model of *L. major*-induced cutaneous leishmaniasis (CL), Tfh cells were detected two weeks post-infection in the draining lymph nodes, where they were required for GC reactions [275]. The transcription factor IRF4 was essential for Tfh cell generation, as *Irf4*-deficient mice completely lacked Tfh cells [275]. This underscores the critical role of IRF4 in Tfh cell differentiation.

Comparative analysis of Tfh responses during *L. infantum*-induced VL in three mouse strains—BALB/c, C57BL/6, and SV/129—revealed strain-specific differences. BALB/c mice were susceptible, whereas C57BL/6 and SV/129 mice were resistant, based on parasite burden and spleen and liver weights [276]. Although CD4 + CXCR5 + PD-1+ Tfh cells were detected in all strains, their presence did not correlate with disease severity [276].

The limited available studies indicate that Tfh cells are generated during *Leishmania spp.* infections and play a role in GC reactions and the production of *Leishmania*-specific antibodies. However, their protective versus pathogenic roles in different forms of leishmaniasis remain unclear. Future research is needed to investigate Tfh cell generation during various *Leishmania spp.* infections and their impact on disease outcomes to better understand their role in leishmaniasis.

## T regulatory (Treg) cells

T regulatory (Treg) cells play a critical role in maintaining self-tolerance by downregulating immune responses to self and non-self antigens. These cells are a subset of CD4+ T cells characterized by the expression of the IL-2 receptor alpha-chain (CD25) and are known to secrete cytokines such as IL-10, and TGF-β [277, 278]. A defining feature of Treg cells is the expression of FOXP3 (forkhead box P3), a member of the forkhead/winged-helix family of transcription factors, which serves as an essential regulator of Treg development and function [279].

Treg cells have been implicated in the persistence of leishmanial parasites in the skin. During *L. major* infection in C57BL/6 mice, CD4 + CD25+ Treg cells accumulate in the dermal layer, accounting for nearly 50% of the CD4+ T cells in the skin [280]. The protective immune response mediated by adoptively transferred *L. major*-immune CD4+ T cells into RAG KO mice was significantly impaired when Treg cells were co-transferred, demonstrating their negative regulatory role in protective T cell responses during *L. major* infection [280]. This regulatory function of Tregs was found to depend on IL-10, as IL-10-deficient Treg cells failed to inhibit protective CD4+ T cell functions [280].

A follow-up study further emphasized the role of IL-10-producing Tregs in parasite reactivation during reinfection at secondary sites [281]. Two key observations were noted: [1] Treg numbers at the primary infection site increased significantly upon reinfection at a secondary site, and [2] this increase correlated with higher swelling, parasite burden, and lesion development at the primary site, even though it was not reinfected. Adoptive transfer of *L. major*-immune Tregs into chronically infected C57BL/6 mice was sufficient to trigger reactivation and increase parasite burden, while Treg depletion during reinfection reduced parasite burden and inhibited reactivation [281]. Consistent with these findings, Zayats et al. demonstrated that *Leishmania*-specific Tregs exhibit higher suppressive activity than polyclonal Tregs, produce IL-10, and facilitate parasite persistence and reactivation [282]. These studies collectively underscore the role of Tregs in suppressing protective immune responses and promoting *L. major* infection and persistence.

Murine Tregs express CCR5 and are responsive to CCR5 ligands *in vitro* [283]. CCR5-deficient C57BL/6 mice displayed increased resistance to *L. major* infection, with reduced lesion size and parasite burden [283]. Notably, the absence of CCR5 impaired Treg homing to infected dermal tissues and significantly reduced IL-10+ T cell populations in lesions, highlighting the importance of CCR5 in Treg-mediated immune suppression [283].

In the context of *L. amazonensis* infection, lesional CD4 + CD25+ Tregs were also observed in C57BL/6 mice. These Tregs suppressed the proliferation and IFN-γ production of conventional CD4+ T cells *in vitro* [284]. Surprisingly, adoptive transfer of Tregs into wild-type (WT) C57BL/6 mice delayed disease onset and reduced lesion size compared to WT mice receiving conventional CD4+ T cells alone, suggesting a protective role for Tregs during *L. amazonensis* infection [284]. Similarly, depletion of Tregs in BALB/c mice using anti-CD25 antibodies (Clone PC61) resulted in more severe lesions with higher parasite loads upon *L. major* infection. This increased susceptibility was associated with a robust IL-4-producing CD4+ T cell response [285]*.* In SCID mice reconstituted with spleen cells from BALB/c mice, Treg depletion also led to greater lesion size and higher parasite burdens compared to controls [285, 286].

In humans, Tregs are more abundant at infection sites than in peripheral blood in patients with cutaneous and mucosal leishmaniasis [287]. This is due to the active migration of Tregs from circulation to the infection site, which is mediated by CCR5 expression. Indeed, the frequency of CCR5-expressing Tregs is significantly higher in infected tissues compared to peripheral blood [287]. In cutaneous leishmaniasis (CL) patients with lupoid and non-lupoid lesions, FOXP3 and IL-10 gene expression levels in PBMCs were significantly higher in lupoid lesions. Importantly, PBMCs from both groups exhibited higher FOXP3 and IL-10 levels than those from healthy subjects [288]. Similarly, skin biopsies from CL patients in Honduras showed increased expression of FOXP3, TGF-β, and IL-10 compared to healthy controls [289]. These human studies suggest a potentially pathogenic role for Tregs in leishmaniasis by facilitating parasite persistence and immune suppression. However, these findings are largely correlative and require further investigation to delineate the precise protective versus pathogenic roles of Tregs during different forms of leishmaniasis.

## CD8 T cells

CD8+ T cells are cytotoxic lymphocytes that recognize peptides presented by major histocompatibility complex I (MHC I), which is expressed by most tissue cells. These cells function by producing pro-inflammatory cytokines (e.g. IFN-γ and TNF) and directly killing infected target cells. Historically, CD8+ T cells were shown to play a protective role during *L. major* infection [232, 290–294]. However, recent studies involving both humans and mice suggest a more pathogenic role for CD8+ T cells [101, 295–301]. The protective and pathogenic roles of CD8+ T cells during leishmaniasis have been extensively reviewed [295]. Here, we provide a general overview of key findings on their protective and pathogenic functions (Fig. 6B).

One of the first studies suggesting a protective role for CD8+ T cells in immunity used thymectomized susceptible BALB/c mice [290]. While depletion of CD8+ T cells did not alter the infection’s course, depletion of CD4+ T cells rendered these mice highly resistant to *L. major* infection [290]. The resistance in CD4+ T cell-depleted BALB/c mice was attributed to increased activity of CD8+ T cells, potentially due to the depletion of Treg cells, which supports a protective role for CD8+ T cells during primary infection [290]. Similar findings were observed in another independent study [292]. Moreover, CD8+ T cells were shown to be crucial for maintaining protective immunity during reinfection [292].

Additionally, CD8+ T cells produce significant amounts of IFN-γ during *L. major* infections in resistant C57BL/6 mice [302]. However, genetic ablation of CD8+ T cells in beta-2-microglobulin knockout (KO) mice and CD8 KO mice—both of which lack functional CD8+ T cells—did not reveal a protective role during *L. major* infection [303, 304]. Both KO mice resolved *L. major* infection at rates comparable to their littermate controls [303, 304]. Given the dominant role of Th1 cells in providing protection during *L. major* infection, it is possible that the protective role of CD8+ T cells—particularly during primary infections—is not as evident when CD4+ T cells are present. Indeed, the protective role of CD8+ T cells was observed only in the absence of CD4+ T cells [290, 292].

Interestingly, Belkaid et al. demonstrated a protective role for CD8+ T cells during primary infection with a low dose of *L. major* (ranging from 100–1000 parasites per infection) [293]. These findings were further validated by Uzonna et al., who directly compared the role of CD8+ T cells in low- versus high-dose *L. major* infections and demonstrated a protective role for CD8+ T cells during low-dose infections [294]**.** Collectively, these studies highlight the protective functions of CD8+ T cells during both primary and secondary infections with *Leishmania* species.

In contrast, more recent studies have demonstrated a pathogenic role for CD8+ T cells. For example, lesion development in *L. major*-infected Rag KO mice—which lack both B and T cells—was exacerbated when these mice were reconstituted with CD8+ T cells but not CD4+ T cells [293, 301]. Notably, while parasite burden was reduced in Rag KO mice receiving CD8+ T cells compared to Rag KO mice alone, lesion size increased, suggesting that CD8+ T cells contribute to parasite control but also drive immunopathology [293]. Similarly, depleting CD8+ T cells in BALB/c mice infected with *L. braziliensis* reduced lesion size but did not affect parasite burden [301].

The pathogenic role of CD8+ T cells has been linked to their cytolytic functions. For instance, perforin-deficient CD8+ T cells—lacking the ability to kill target cells—did not induce larger lesions compared to wild-type CD8+ T cells [301]. Several mechanisms have been proposed for CD8+ T cell-mediated immunopathology during *Leishmania spp.* infections. These include roles for the NKG2D receptor [297, 305, 306], inflammasome activation and IL-1β production [300], and, more recently, CCR5 signaling [296].

Given that macrophages serve as the primary host cells for *Leishmania spp.*, the cytotoxic activity of CD8+ T cells may play a role in controlling infection. However, this cytolytic function can also have unintended consequences, leading to tissue damage and lesion development independent of parasite control.

## B cells

**Figure 7:**
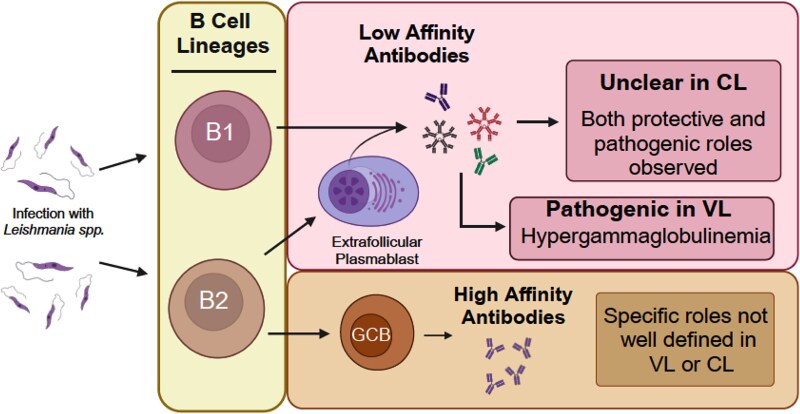
B cells and antibodies in leishmaniasis. B cells play critical roles during leishmania infections, however the mechanistic details of how this occurs are still being elucidated. B1 cells have been shown to play both protective and pathogenic roles in CL, and pathogenic roles in VL. Low affinity antibodies produced by B1 cells and extrafollicular plasmablasts have been shown to be pathogenic in VL, but specific roles are unclear in CL. B2 cells are involved in germinal center reactions to produce high affinity antibodies, however, their roles are not well defined in VL or CL.

B cells have long been studied for their production of highly specific antibodies that neutralize pathogens, as well as their role as professional APC [307, 308]. Infections with trypanosome parasites specifically manipulate B cells to promote parasite persistence and establish chronic infection [308]. While much data supports the critical role of B cells in the immune response during leishmaniasis, these responses have historically been understudied (Fig. 7). This may be due, in part, to contradictory data that points to both pathogenic and protective roles for B cells in leishmaniasis [309, 310]. These paradoxical roles have been attributed to differences between parasite species, genetic backgrounds, and, more recently, the lineage of B cells [307, 309, 311]. Emerging work suggests that distinguishing between innate-like B1 cells, which produce low specificity, polyreactive antibodies, and affinity-matured B2 cells, which produce high specificity antibodies, may provide further clarity on their roles [307, 309, 311–313].

One example of this paradox is observed in *L. amazonesis* infection, where antibodies assist in macrophage killing of parasites but simultaneously promote the migration and priming of IL-10-producing CD4+ T cells [313, 314]. IL-10 production is associated with decreased production of IFN-γ and nitric oxide, both of which are essential for efficient macrophage killing of parasites [313, 315, 316]. Several studies have linked Th2 responses and IL-10 production to the pathogenesis of leishmaniasis [317–319]. B1 cells are known to be major producers of IL-10, but their contribution to IL-10-mediated pathology in *Leishmania spp.* infection has only recently been explored [311, 312].

Counterintuitively, there is clear evidence that B1 cells positively impact *L. amazonesis*-mediated CL in mice on a susceptible BALB/c genetic background. When infected, BALB/XiD mice—which have a mutation in Bruton’s Tyrosine Kinase (BTK) that results in a drastic reduction of B1 cells and impaired maturation of B2 cells—exhibited higher parasite burdens, decreased anti-leishmania IgM, and an increase in IL-10 within the spleen [311]. When B1 cells from wild-type (WT) BALB/c mice were transferred into BALB/XiD mice and infected, susceptibility was significantly reduced. This result aligns with previous studies using B cell deletion models, but surprisingly, it also led to an increase in splenic IL-10 in BALB/XiD mice [309]. This IL-10 likely derives from other cell types, suggesting that pathogenic IL-10 production is not from B1 cells.

Furthermore, it has been shown that B1 cells can phagocytose *L. amazonesis* parasites both *in vivo* and *in vitro*, which significantly impacts their cytokine production [312]. Although infection did not seem to influence IL-10 production *in vitro*, qRT-PCR of mRNA from total B1 cells isolated from infected mice revealed a significant increase in IL-10 expression. Interestingly, the amount of IL-10 produced by peritoneal macrophages isolated from the same mice did not significantly differ between infected and uninfected mice [312]. This suggests that IL-10 production by B1 cells is not mediated by intracellular infection but is instead induced extracellularly. Additionally, there was an increase in TNF production by infected B1 cells both *in vitro* and *in vivo* [312]. TNF is secreted by macrophages as they activate to kill parasites, but its role in pathogenesis or protection differs between and CL and VL [320, 321]. Taken together, these findings point to an interesting phenotypic bifurcation of B1 cells in *L. amazonesis* infection that could influence healing, but further investigation is needed.

One mechanism that could influence the phenotypic bifurcation of B1 cells in *L. amazonesis* infection is the release of extracellular vesicles (EVs) by infected B1 cells [322, 323]. When B1 cells isolated from BALB/c mice were infected with *L. amazonesis*, they produced a significantly higher amount of EVs [323]. These EVs have been shown to alter cytokine expression in naïve macrophages derived from both BALB/c and C57BL6 mice [323–325]. C57BL6-derived macrophages treated with EVs from infected B1 cells exhibited a significant increase in TNF-α production, a significant decrease in IL-10 production, and a notable decrease in iNOS production [323]. In contrast, BALB/c-derived macrophages did not show changes in iNOS or TNF-α production but exhibited a significant increase in IL-10 production. When EVs from both uninfected and infected B1 cells were administered *in vivo*, the lesion size in BALB/c mice was significantly reduced. Furthermore, the parasite burden in both groups treated with EVs was significantly lower compared to untreated controls, with the group treated with EVs from uninfected B1 cells also showing reduced parasite loads [323]. This suggests that the phenotype of B1 cells may influence the resistance or susceptibility of the mouse model and highlights the role of B1 cells beyond antibody production.

The role of high-affinity antibodies in leishmaniasis remains an area that has not been thoroughly investigated, although low-affinity antibody production has been linked to disease severity and parasite persistence. IRF-4 deficiency in mice has been associated with higher susceptibility to *L. major* infection, as well as a reduction in germinal center B cell numbers within the draining lymph nodes [275]. Upregulation of IRF-4 has recently been connected to TSLPR signaling in both B and T cells, as well as the formation of germinal centers [326]. This is particularly interesting because, as discussed in the eosinophil section, tissue-resident macrophages in *L. major* infection have been shown to produce TSLP, which has been implicated in early infection signaling with ILC2s and eosinophils [57]. This suggests that TSLP production during *L. major* infection may also directly impact germinal center formation and the production of high-affinity antibodies.

As mentioned earlier in this review, opsonization of *Leishmania spp.* with IgG antibodies can influence parasite internalization and phagolysosome maturation [119, 121]. In patients with VL, hypergammaglobulinemia (HGG) is a key diagnostic characteristic and predictor of disease severity [327, 328]. HGG refers to the overproduction of multiple classes of low-specificity antibodies [329]. It has been well documented that during *Leishmania* infection, there is polyclonal B cell activation, leading predominantly to low-affinity anti-*Leishmania* antibodies and increased circulating immune complexes [328, 330, 331]. As a Th2 cytokine, IL-10 has been implicated in blocking Th1-mediated inflammation in both VL and CL [332]. In addition to being associated with increased disease severity, Th2 cytokines have been shown to stimulate B cell proliferation and differentiation more effectively than Th1 cytokines [333, 334]. In fact, the therapeutic effectiveness of treatments given to VL patients who later develop post-kala-azar dermal leishmaniasis (PKDL) is monitored via IL-10 and IgG antibody production [335].

Taken together, these findings suggest that B cells and antibodies play disparate roles in the immune response to leishmaniasis. Despite this, B cell depletion in the context of CL induced by *Leishmania* spp. does not appear to prevent mice from effectively combating the infection, and B cell-deficient mice are reported to be highly resistant to experimental VL [309, 336]. This suggests that while B2 cells, the dominant population, likely play a primarily pathogenic role in both CL and VL, B1 cells may contribute to the conflicting data and paradoxical understanding of B cells in leishmaniasis.

## Conclusion and perspective

*Leishmania* parasites are obligate intracellular pathogens that affect over a billion people worldwide. During infection, the parasite encounters a variety of immune and non-immune cells, all of which play critical roles in shaping the course of infection and disease progression. Macrophages, as sentinel cells, serve as the reservoirs for *Leishmania* parasites, providing a niche for them to hide and propagate. However, several other immune and non-immune cells are also known to be parasitized by *Leishmania*. Keratinocytes, neutrophils, and dendritic cells have all been shown to be positive for intracellular *Leishmania* and may serve as alternative reservoirs for these parasites. Additionally, our knowledge of how adaptive immune cells—particularly T and B cells—cooperate to provide protection during *Leishmania* infection has significantly improved. We now have a more detailed understanding of how different subsets of T cells function during *Leishmania* infections.

Despite these advancements, there remains considerable ambiguity regarding how various cell types contribute to *Leishmania* infections. The disease course and pathology vary greatly depending on the infecting parasite species, the vector transmitting the parasite, and the host. Consequently, extrapolating and generalizing findings from one model system to another can present significant limitations. Moreover, most studies tend to focus on immune responses from the perspective of a single cell type, which can sometimes overstate the impact of that particular cell type in the disease process. It is important to recognize the significant redundancy within the immune system, including the diverse cell types and immune responses activated against *Leishmania* parasites. This, combined with *Leishmania*’s ability to hijack host immune responses—particularly innate immune cells from within—poses a significant challenge in the development of effective therapeutics [337]. More comprehensive research examining holistic immune responses across multiple *Leishmania* species will be necessary to resolve conflicting findings and to develop novel immune-based strategies for combating leishmaniasis.
